# Hydrogen sulfide-induced itch requires activation of Ca_v_3.2 T-type calcium channel in mice

**DOI:** 10.1038/srep16768

**Published:** 2015-11-25

**Authors:** Xue-Long Wang, Bin Tian, Ya Huang, Xiao-Yan Peng, Li-Hua Chen, Jun-Cheng Li, Tong Liu

**Affiliations:** 1 Jiangsu Key Laboratory of Translational Research and Therapy for Neuro-Psycho-Diseases and the Second Affiliated Hospital of Soochow University, Soochow University, Suzhou 215021, China; 2 Institute of Neuroscience, Soochow University, Suzhou, Jiangsu, 215123, China; 3 Jiangsu Key Laboratory of Preventive and Translational Medicine for Geriatric Diseases, School of Public Health, Soochow University, Suzhou, 215123, China

## Abstract

The contributions of gasotransmitters to itch sensation are largely unknown. In this study, we aimed to investigate the roles of hydrogen sulfide (H_2_S), a ubiquitous gasotransmitter, in itch signaling. We found that intradermal injection of H_2_S donors NaHS or Na_2_S, but not GYY4137 (a slow-releasing H_2_S donor), dose-dependently induced scratching behavior in a μ-opioid receptor-dependent and histamine-independent manner in mice. Interestingly, NaHS induced itch via unique mechanisms that involved capsaicin-insensitive A-fibers, but not TRPV1-expressing C-fibers that are traditionally considered for mediating itch, revealed by depletion of TRPV1-expressing C-fibers by systemic resiniferatoxin treatment. Moreover, local application of capsaizapine (TRPV1 blocker) or HC-030031 (TRPA1 blocker) had no effects on NaHS-evoked scratching. Strikingly, pharmacological blockade and silencing of Ca_v_3.2 T-type calcium channel by mibefradil, ascorbic acid, zinc chloride or Ca_v_3.2 siRNA dramatically decreased NaHS-evoked scratching. NaHS induced robust alloknesis (touch-evoked itch), which was inhibited by T-type calcium channels blocker mibefradil. Compound 48/80-induced itch was enhanced by an endogenous precursor of H_2_S (L-cysteine) but attenuated by inhibitors of H_2_S-producing enzymes cystathionine γ-lyase and cystathionine β-synthase. These results indicated that H_2_S, as a novel nonhistaminergic itch mediator, may activates Ca_v_3.2 T-type calcium channel, probably located at A-fibers, to induce scratching and alloknesis in mice.

Itch (pruritus) is an unpleasant cutaneous sensation that elicits scratch reflex^1^. Although itch and pain share many similarities, recent studies revealed that itch has its own unique molecular, cellular and circuitry mechanisms^2^. Acute itch serves as a self-protective mechanism to prevent our bodies from harmful external irritants^1^. However, chronic itch is a debilitating symptom that accompanies numerous skin and systemic diseases, including atopic dermatitis and psoriasis, chronic kidney failure and cholestasis, diabetes and some cancer^3^. Antihistamines are the first choice for treating allergic itch. However, they are inefficient for many other chronic itch conditions^4^, suggesting that histamine-independent mechanisms are involved in^2^. Although itch sensation can be transiently relieved by scratching^5^, itch-scratch-itch cycles often exacerbate skin problems^6^. Chronic itch disrupts sleep and substantially reduces the quality of life of patients. Thus, there is an urgency to identify novel non-histaminergic itch mediators, which may be involved in the pathogenesis of chronic itch.

Hydrogen sulfide (H_2_S) is now considered to be the third gasotransmitter in addition to nitric oxide (NO) and carbon monoxide (CO)^7^. H_2_S is endogenous produced enzymatically mainly by cystathionine γ-lyase (CSE) and cystathionine β-synthase (CBS) from L-cysteine or by 3-mercaptopyruvate sulfurtransferase (MPST) with cysteine aminotransferase (CAT) from 3-mercaptopyruvate^8^. In recent years, H_2_S is becoming a molecule of high interest and getting more attention to its physiological and pathological functions involved in the regulation of cardiovascular system, nervous system, gastrointestinal system, renal function and inflammatory responses^8^,^9^,^10^,^11^,^12^,^13^. Increasing evidence supports that H_2_S is involved in modulation of pain processing^14^,^15^. Although itch and pain are distinct sensations and have separate neural pathways^2^, they might share similar mediators and receptors^16^. Interestingly, recent studies emphasized the crucial contribution of NO in itch signaling elicited by chloroquine, serotonin and substance P^17^,^18^,^19^, suggesting an important role of gasotransmitter in itch signaling. In sharp contrast, the roles of H_2_S in itch regulation remain elusive.

The aim of the present study is to test whether H_2_S can induce itch behaviors in mice and further elucidate the underlying molecular mechanisms. Itch behavior can be quantitatively evaluated by measuring the scratching behavior elicited by pruritogens and can be differentiated from pain by using cheek model in rodents^20^,^21^. In this study, we investigated the behavioral responses in mice induced by intradermal (i.d.) injection of NaHS or Na_2_S, two commonly used H_2_S donors. We firstly found that H_2_S could elicit robust scratching behavior, which required activation of Ca_v_3.2 T-type calcium channel, but not TRPV1 and TRPA1. In contrast, H_2_S-induced pain required activation of both T-type calcium channel and TRPV1. We next revealed that endogenous production of H_2_S contributes to compound 48/80-induced itch sensation by using CBS inhibitor aminooxyacetic acid (AOAA) and CSE inhibitor dl-Propargylglycine (PAG). Thus, our results identified H_2_S as a novel itch mediator and indicated Ca_v_3.2 T-type calcium channel inhibitors or H_2_S synthesis inhibitors may be novel promising strategies for management of itch, although the precise roles of H_2_S in chronic itch need further investigation.

## Materials and Methods

### Animals

Adult male CD1 mice (8–10 weeks old upon arrival) used in this study were obtained from Laboratory Animal Center of Chinese Academy of Sciences. Animals were housed with food and water available *ad libitum* and kept in controlled room temperature (22 ± 2 °C) and humidity (60–80%) under a 12 h/12 h light/dark cycle. All the behavioral tests were done in blind respect to the drug treatment. All experimental procedures and animal handing were performed in accordance with the guidelines of the International Association for the Study of Pain and the animal protocols were approved by Soochow University Animal Committee. The authors tried all efforts to minimize the number of animals used.

### Drugs and administration

We purchased NaHS, Na_2_S, GYY4137, compound 48/80, chloroquine, Resiniferatoxin (RTX), chlorpheniramine maleate, and capsaicin from Sigma-Aldrich (St. Louis, MO, USA). Zinc chloride was obtained from China Sinopharm Chemical Reagent CO.,Ltd (Shanghai, China), and ascorbic acid was obtained from China Shanghai Xiandai Hasen (Shangqiu) Pharmaceutical CO.,Ltd (Shanghai, China). Naloxone hydrochloride was obtained from China National Medicines Guorui Pharmaceutical CO.,Ltd (Huainan City, Anhui Province, China). Mibefradil dihydrochloride, capsazepine and HC-030031 was obtained from Tocris (Bristol, UK). Morphine hydrochloride was obtained from China Northeast Pharmaceutical Group Shenyang No.1 Pharmaceytical CO., Ltd (Shenyang City, Liaoning Province, China). lidocaine(2%) was obtained from China Otsuka Pharmaceytical CO.,Ltd (Tianjin, China). Capsaicin, Resiniferatoxin (RTX), capsazepine, mibefradil and HC-030031 were dissolved in 10% DMSO. Other reagents were dissolved in sterile saline if not specified.

Selective Ca_v_3.2 siRNA (sc-42707) and scrambled Ca_v_3.2 control siRNA (sc-37007) were synthesized by Santa Cruz (Shanghai, China). siRNA was dissolved in RNase-free water at the concentration of 1 μg/μl as stock solution, and mixed with polyethyleneimine (PEI, Fermentas) (Shanghai, China), 10 min before injection, to increase cell membrane penetration and reduce the degradation. PEI was dissolved in 5% glucose, and 1 μg of siRNA was mixed with 0.18 μl of PEI. We intrathecally injected 10 μl of siRNA (2 μg) once a day for 2 days to knockdown Ca_v_3.2 expression. Intrathecal injection was performed by a lumbar puncture to deliver reagent into cerebral spinal fluid. A successful spinal puncture was evidenced by a brisk tail-flick after the needle entry into subarachnoid space^22^. Capsaicin (10 μg in 20 μl 2.5% DMSO) or NaHS (200 μg in 20 μl saline) was intraplantarly injected into one hindpaw, and the number of flinches was counted for the first 10 min.

### Neck model of itch

As described previously^23^, mice were shaved at the nape of the neck at least 2 day before experiments. On the day of behavioral testing, mice were individually placed in small plastic chambers (10 × 10 × 12.5 cm) on an elevated metal mesh floor and allowed at least 30 min for habituation. Under brief anesthesia of isoflurane, mice were given an intradermal injection of 50 μl of agents via a 26G needle into the nape of the neck. Immediately after the injection, mice were returned to their chambers and were video recorded for 30 min. The video was subsequently played back offline and itch behavior was quantified by counting the number of scratches in a blinded manner. A scratch was counted when a mouse lifted its hindpaw to scratch the shaved region and returned the paw to the floor or to the mouth for licking.

### Cheek model of itch

To distinguish itch and pain behavior, we used the cheek model by injection of chemicals into the cheek of mice. Mice were shaved on cheeks (approx. 5 × 8 mm area) at least 2 day before the experiment. On the day of experiment, mice were intradermally injected of 20 μl of reagent (NaHS or Na_2_S) via a 26G needle into the cheek under brief anesthesia with isoflurane. Immediately after the injection, mice were returned to their chambers and were video recorded for 30 min. The video was played back and the number of wipes and scratches were quantified by counting their number. One wipe was defined when mouse unilaterally wipes the injected site with the forelimb, which was not part of grooming behavior. One scratch was defined as a lifting of the hind paw toward the injection site on the cheek and then returning the paw to the floor or to the mouth.

### Alloknesis assay

According to a previous report^24^, alloknesis after intradermal injection of pruritogens was evaluated. Briefly, 30 min after the injection of NaHS or Na_2_S, a von Frey filament (0.7 mN) was applied to the affected skin site. A scratch bout directed to the site of mechanical stimulation was considered as a positive response. The alloknesis score was determined by calculating the total number of scratches elicited by three mechanical stimuli and was evaluated at 5-min intervals.

### Tail immersion test

As previously described^25^, tail immersion test was employed to assess heat pain sensitivity in mice. Briefly, the terminal 3 cm of a mouse’s tail was immersed in hot water bath at 52 °C and the latency of tail flick was recorded with a cutoff time of 10 seconds to avoid potential tissue injury.

### Rotarod test

The mouse motor function was tested using DXP-2 Rota-Rod equipment (Institute of Materia Medica, Chinese Academy of Medical Sciences). Each mouse was trained for two consecutive days (6 trails per day) where the speed of the rotor was accelerated from 4 to 25 rpm with an acceleration of 0.2 rpm/sec. One day after the last training session, the mouse was tested at the speed of the rotor (25 rpm) for three times and the longest duration of running time was recorded.

### Pharmacological treatments

To test the possible effects of μ-opioid receptor agonist or antagonist on H_2_S donors-induced scratching, μ-opioid receptor agonist morphine (1 mg/kg) or antagonist naloxone (1 mg/kg) was i.p. injected into mice 20 min before i.d. injection of 200 μg NaHS or Na_2_S in mice. To assess the possible effects of antihistamine on H_2_S donors-induced scratching behavior, chlorpheniramine (10 mg/kg), the selective histamine H1 receptor antagonist, was i.p. injected 20 min before i.d. injection of NaHS or Na_2_S in mice. To examine the role of mast cells in the H_2_S donors-induced itch, we deplete mast cells by daily treatment with compound 48/80 (1, 3, 10 and 10 μg per site on the 1st, 2nd, 3rd and 4th days, respectively) before injection of NaHS. As we described previously^23^, to examine the role of TRPV1-expressing C-fibers in the H_2_S donors-induced itch, we destroyed these C-fibers by daily treatment with the ultra-potent TRPV1 receptor agonist resiniferatoxin (RTX, 30, 70 and 100 μg/kg, subcutaneously for 3 consecutive days), one week before injection of H_2_S donors. Intradermal injection of lidocaine (2%) was also used for testing the possible role of A-fibers in H_2_S donors-induced scratching in both vehicle and RTX-treated mice^26^. To assess the involvement of TRPV1 or TRPA1 in H_2_S donors-induced scratching behavior, capsazepine (10–50 μg; the selective TRPV1 antagonist) or HC-030031 (10–50 μg; the selective TRPA1 antagonist) was intradermally co-administrated with NaHS. To assess the involvement of Ca_v_3.2 T-type calcium channel in NaHS-induced scratching behavior, mibefradil (i.d. 5–25 nmol; T-type calcium channel blocker), ascorbic acid (i.d. 1 nmol; i.p. 1 mg/kg; selective Ca_v_3.2 blocker) or zinc chloride (i.d. 5 nmol; i.p. 1 mg/kg; selective Ca_v_3.2 blocker) were administrated. The doses chosen for inhibition experiments were based on previous reports and our pilot experiments.

### Real-time quantitative RT-PCR

We collected cervical DRGs and spinal cord, isolated total RNAs using RNeasy Plus Mini kit (Qiagen, Valencia, CA). One microgram of RNA was reverse transcribed for each sample using Omniscript reverse transcriptase according to the protocol of the manufacturer (Qiagen). Q-RT-PCR sequences for Ca_v_3.2: forward: TCTCGCTACCCAATGACAGC; reverse: CTCCGTGTAGTCTGGGATGC; Ca_v_3.1: forward: ACATTGGAGCAGCCTCTTCAG; reverse: CTGCTGGTTGGGAGTGAACA. Triplicate qPCR analyses were performed using the SYBR Green master mix (KAPA) and Opticon real-time PCR Detection System (ABI Life7500) as described previously^27^.

### Immunohistochemistry

Mice were terminally anesthetized with isoflurane and perfused through the ascending aorta with saline followed by 4% paraformaldehyde. The L4-6 lumbar spinal cords were collected and postfixed in the same fixative overnight. The spinal cord sections were cut at the thickness of 14-μm in a cryostat. The tissue sections were blocked with 10% goat serum, and incubated over night at 4^o^C with the primary antibodies guinea pig anti-TRPV1 antibody (1:1000, Neuromics) (Edina, MN, USA). The sections were then incubated for 1 h at room temperature with Cy3-conjugated secondary antibodies (Jackson ImmunoResearch. West Grove, PA, USA). Immunostained tissue sections were examined under a Zeiss fluorescence microscope AXIO SCOPE A1 (Oberkochen, Germany), and images were analyzed with NIH Image software or Adobe PhotoShop.

### Western blotting

Mice were terminally anesthetized with isoflurane and transcardially perfused with PBS, and the DRGs were rapidly removed and homogenized in a lysis buffer containing a cocktail of protease inhibitors and phosphatase inhibitors for total protein extraction and assay according to our previous report^28^. The protein concentrations were determined by BCA Protein Assay (Pierce, Rockford, IL, USA), and 25 μg of proteins were loaded for each lane and subjected to SDS-PAGE. After the transfer, the blots were blocked with 5% nonfat milk in TBST and PVDF membranes were incubated overnight at 4°C with primary polyclonal antibody against Ca_v_3.2 (goat, 1:500; Santa Cruz Biotechnology). For loading control, the blots were probed with Tubulin antibody (mouse, 1:1000, Vazyme, Suzhou City Jiangsu Province, China). The blots were washed and incubated in horseradish peroxidase–conjugated donkey anti-goat or goat anti-mouse IgG secondary antibody (1:2000, Santa Cruz Biotechnology). Protein bands were visualized using an enhanced chemiluminescence detection kit (Pierce) and the band densities were detected and analyzed using Molecular Imager ChemiDoc XRS + System (Bio-Rad, Shanghai, China). Data from five mice were used for statistical analysis.

### Statistical analysis

All values were presented as the mean ± S.E.M. Student’s *t* test was used for two group comparison. One-way ANOVA followed by post-hoc Bonferroni test was used for multiple comparisons. Two-way repeated-measured ANOVA was also used to analyze the data with multiple time points. Differences with *p* < 0.05 were considered as statistical significance.

## Results

### Intradermal injections of H_2_S donors induce scratching behavior in mice

**Figure 1:**
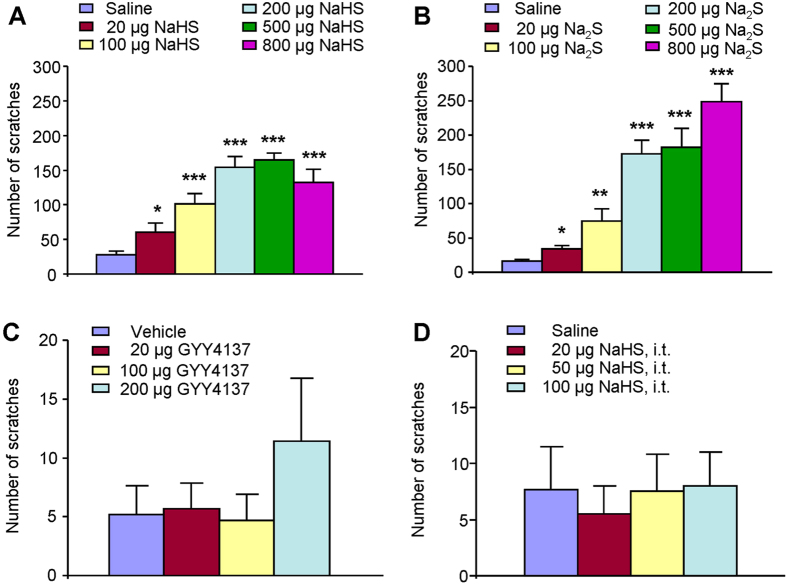
Scratching behavior induced by H_2_S donors in mice. (**A,B**) Dose-dependent scratching behavior induced by intradermal (i.d.) injection of H_2_S donors NaHS (20–800 μg; A) or Na_2_S (20–800 μg; B). (**C**) No obvious scratching behavior induced by i.d. GYY4137, a slow-releasing H_**2**_S donor. (**D**) No obvious scratching behavior induced by intrathecal injection of NaHS (20–100 μg). Saline injection (50 μl, i.d) serves as control. All data are expressed by means ± SEM. *n* = 6–8 mice per group. *P < 0.05, **P < 0.01; ***P < 0.001 vs. control, one-way AVOVA following Bonferroni *post hoc* test.

We firstly investigated whether intradermal (i.d.) injection of NaHS and Na_2_S, two commonly used H_2_S donors, are able to induce scratching behavior in CD1 mice. Injection of NaHS (i.d., 20–800 μg; Fig. 1A) or Na_2_S (i.d., 20–800 μg; Fig. 1B) into the nape of neck produced robust scratching behavior in a dose-dependent manner in mice. NaHS or Na_2_S began to elicit scratching at the dose of 20 μg and reached a platform at the doses of 200–800 μg. Thus, the dose of 200 μg NaHS or Na_2_S was chosen for the following experiments. We did not observe any abnormal behaviors of mice following i.d. application of H_2_S donors in these experiments. In contrast, injection of GYY4137 (i.d., 20–200 μg), a slow-releasing H_2_S donor, into the nape of neck did not produced obvious scratching in mice (Fig. 1C). We also found that intrathecal (i.t.) injection of NaHS (20–100 μg) did not elicited obvious scratching (Fig. 1D), indicating peripheral, but not central, application of H_2_S donors elicit itch in mice.

**Figure 2:**
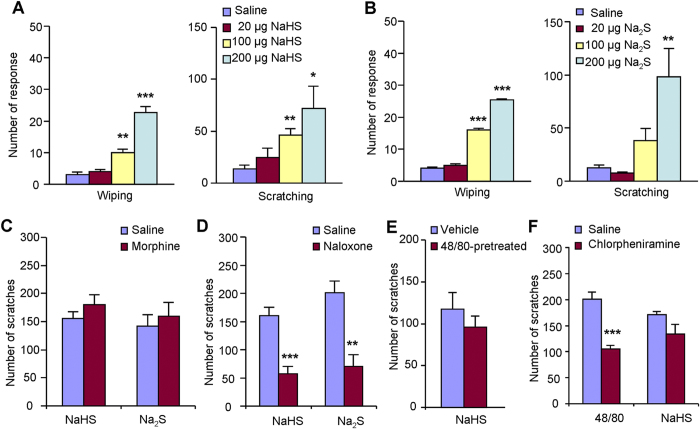
Pharmacological interventions of H_2_S donors-induced scratching behavior in mice. (**A**,**B**) Both forelimb wiping and hindpaw scratching could be induced by i.d. 20 μl NaHS (20–200 μg; A) or Na_2_S (20–200 μg; B) into cheek, indicating H_2_S donors-induced mixed pain and itch sensation. i.d injection of 20 μl saline served as a control. *P < 0.05, **P < 0.01; ***P < 0.001 vs. control, one-way AVOVA following Bonferroni *post hoc* test. (**C,D**) Naloxone (i.p. 1 mg/kg; **D**) but not morphine (i.p. 1 mg/kg; **C**) significantly reduced NaHS or Na_2_S-induced scratching behavior. **P < 0.01; ***P < 0.001 vs. control, Student’s *t* test. (**E**) In compound 48/80-pretreated mice, NaHS (200 μg) could induce comparable scratching behavior, suggesting mast cells were not critically involved in. (**F**) Systemic injection of chlorpheniramine (a histamine H1 antagonist; 10 mg/kg, i.p.) suppressed scratching behavior induced by compound 48/80 (100 μg) but not by NaHS (200 μg), suggesting histamine-independent mechanisms were involved in. ***P < 0.001; vs. control, Student’s *t* test. All data are expressed by means ± SEM. *n* = 5–8 mice per group.

In order to distinguish itch and pain behaviors in rodents, we used the cheek model by i.d. injection of chemicals into cheek of mice^29^. In cheek model, painful agents elicit forelimb wiping behavior, while itchy agents elicit hindlimb scratching behavior^29^. I.d. injection of NaHS (Fig. 2A) or Na_2_S (Fig. 2B) into the cheek of mice dose-dependently elicited both wiping and scratching behavior, indicating these H_2_S donors can induce mixed itch and pain sensations. Notably, both NaHS and Na_2_S induced relative more scratching than wiping behavior, suggesting that itch may be one of the major sensory modalities induced by these H_2_S donors.

### Intradermal injections of H_2_S donors induce scratching in an opioid receptor-dependent and histamine-independent manner in mice

We next tested whether H_2_S donors-induced scratching could be modulated by μ-opioid receptor, which has been implicated in itch for rodents and humans^30^. Morphine, a μ-opioid receptor agonist, is clinical used as analgesic^31^. Systemic morphine (1 mg/kg, i.p.) did not reduce NaHS or Na_2_S-induced scratching behavior (Fig. 2C), consistent with clinical observations that morphine did not reduce even exacerbate itch^31^. However, naloxone (1 mg/kg, i.p.), an opioid receptor antagonist, significantly attenuated scratching induced by NaHS (160.1 ± 15.2 vs. 57.4 ± 12.5; *P* < 0.001; Fig. 2D) or Na_2_S (201.0 ± 20.5 vs. 70.1 ± 20.9; *P* < 0.001; Fig. 2D). The result indicated that NaHS or Na_2_S-induced scratching was itch-related behavior in mice and also suggested that endogenous opioids may be involved in H_2_S donors-induced itch in mice.

One of the best-known itch mediators is histamine, which is stocked and released from skin cells, such as mast cells and keratinocytes^32^. We subsequently asked whether mast cells and histamine were involved in H_2_S donors-induced scratching in mice. Mice were pretreated with compound 48/80 to cause skin mast cells degranulation and reduce the number of mast cells^33^. It was found that NaHS induced comparable scratching in vehicle and compound 48/80-pretreatment mice (Fig. 2E), suggesting mast cells have little effects on NaHS-induced itch. Systemic injection of histamine H1 receptor antagonist chlorpheniramine (10 mg/kg, i.p.) did not affect NaHS-induced scratching in mice (Fig. 2F), but significantly decreased compound 48/80-induced scratching in mice (200.8 ± 13.4 vs. 105.3 ± 7.0; *P* < 0.001; Fig. 2F). Thus, H_2_S donors-elicited scratching behavior is largely independent of mast cells and histamine.

### TRPV1-expressing C-fibers are dispensable for H_2_S donors-induced scratching behavior in mice

**Figure 3:**
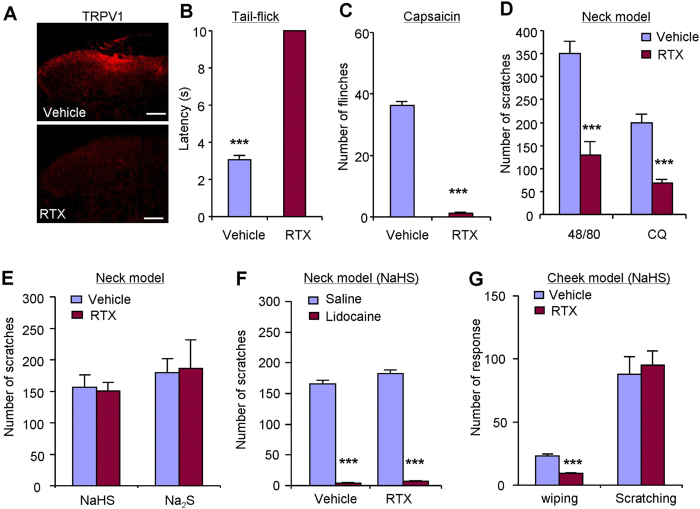
TRPV1-expressing C-fibers were not required for H_2_S donors-induced itch behaviors in mice. (**A**) Immunostaining showing that TRPV1-expressing C-fibers are dramatically reduced in RTX-treated mice comparing with vehicle-treated mice. (**B**) Tail-flick latency of RTX-treated mice reached cutoff time (10 s) in 52 °C hot water bath. ***P < 0.001 vs. control, Student’s *t* test. (**C**) Flinches induced by capsaicin (10 μg) were abolished in RTX-treated mice. (**D**) Compound 48/80 or chloroquine-induced scratching were significantly inhibited in RTX-treated mice. (**E**) NaHS- or Na_2_S-induced scratching behavior was comparable in vehicle- and RTX-treated mice. (**F**) Local application of lidocaine (2%) completely abolished NaHS-induced scratching in both vehicle- and RTX-treated mice. (**G**) In cheek model, NaHS-induced forelimb wiping, but not hindpaw scratching, was abolished in RTX-treated mice, suggesting capsaicin-sensitive C-fibers were required for NaHS-induced pain but not itch. All data are expressed by means ± SEM. n = 6–9 mice per group. ***P < 0.05 vs. control, Student’s *t* test.

It is well appreciated that TRPV1-expressing C-fibers mediate itch sensation induced by histaminergic and nonhistaminergic pruritogens^34^,^35^. To further determine whether TRPV1-expressing C-fibers mediate H_2_S donors-induced itch, we employed systemic pretreatment of resiniferatoxin (RTX), an ultrapotent TRPV1 agonist, to destroy TRPV1-expressing C-fibers in mice. Consistent with our previous work, immunostaining showed that pretreatment with RTX resulted in lack of TRPV1-positive signals in spinal cord dorsal horn of mice (Fig. 3A). The tail-flick latency of 52 °C noxious heat reached to cutoff time in RTX-treated mice (Fig. 3B). Intraplantar capsaicin-induced flinching was abolished in RTX-treated mice (36.3 ± 1.5 vs. 1.3 ± 0.4; *P* < 0.001; Fig. 3C). Together, these data confirmed the loss of TRPV1-expressing C-fibers in mice after RTX treatment. As expected, scratching induced by compound 48/80 or chloroquine (CQ) were dramatically reduced in RTX-treated mice compared with control mice (Fig. 3D). In sharp contrast, NaHS or Na_2_S-induced scratching was comparable between vehicle and RTX-treated mice (Fig. 3E). These data suggested TRPV1-expressing C-fibers are dispensable for H_2_S donors-induced itch in mice.

To further define the roles of A-fibers in NaHS-induced itch, local injection lidocaine (2%), which is a local anesthetic blocked both A- and C-fibers, completely abolished NaHS-induced scratching in both vehicle (165.7 ± 6.5 vs. 4.2 ± 0.6; *P* < 0.001; Fig. 3F) and RTX-treated mice (182.7 ± 5.5 vs. 7.0 ± 1.1; *P* < 0.001; Fig. 3F). In cheek model, NaHS-induced scratching was also not affected by RTX treatment, while NaHS-induced wiping was significantly reduced in RTX-treated mice (Fig. 3G). Thus, it suggested capsaicin-insensitive A-fibers might mediate H_2_S donors-induced itch, while capsaicin-sensitive C-fibers mediated H_2_S donors-induced pain in mice.

### Neither TRPV1 nor TRPA1 is required for H_2_S donors-induced itch in mice

**Figure 4:**
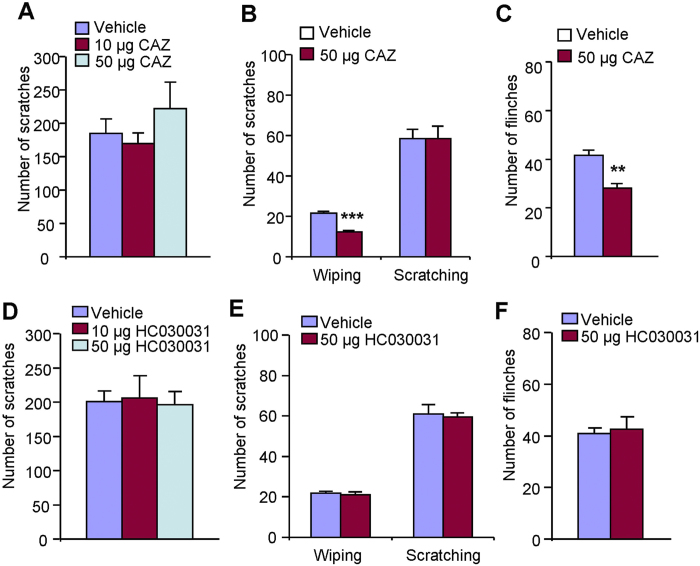
TRPV1 and TRPA1 were not required for NaHS-induced itch, while TRPV1 was required for NaHS-induced pain in mice. (**A**) Local administration of TRPV1 antagonist capsazepine (10–50 μg) did not affect NaHS-induced scratching. (**B**) In cheek model, co-administration of capsaizepine (50 μg) suppressed NaHS-induced forelimb wiping, but not hindpaw scratching. (**C**) Intraplantar co-administration of capsazepine (50 μg) attenuated NaHS-induced flinching. (**D**) Local administration of TRPA1 antagonist HC-030031 (10–50 μg) did not affect NaHS-induced scratching. (**E**) In cheek model, co-administration of HC-030031 (50 μg) did not affect NaHS-induced both forelimb wiping and hindpaw scratching. (**F**) Intraplantar co-administration of HC-030031 (50 μg) had no effects on NaHS-induced flinching. All data are expressed by means ± SEM. *n* = 6–8 mice per group. *P < 0.05; **P < 0.01, ***P < 0.001 vs. vehicle control, Student’s *t* test.

TRPV1 and TRPA1 were well-demonstrated to mediate histamine-dependent and –independent itch, respectively, in mice^35^,^36^. Recent work showed that excitatory effect of H_2_S on gastrointestinal motility may be attributed to the activation of TRPV1^37^. H_2_S-induced mechanical pain is also partially mediated by the activation of TRPA1 in mice^38^. Thus, it suggested H_2_S may act on TRPV1 and/or TRPA1 to produce pain in mice. In this study, we subsequently examined the role of TRPV1 and TRPA1 in H_2_S donors-induced itch using pharmacological methods. Co-administration of TRPV1 selective blocker capsazepine (10–50 μg) in either neck or cheek did not affect NaHS-induced scratching in mice (Fig. 4A,B). Capsazepine (50 μg) significantly reduced intradermal NaHS-induced forelimb wiping (21.6 ± 0.8 vs. 12.2 ± 0.7; *P* < 0.001; Fig. 4B) and intraplantar NaHS-induced flinching in mice (41.6 ± 2.1 vs. 28.0 ± 2.1; *P* < 0.001; Fig. 4C). Co-administration of HC-030031 (10–50 μg), a selective TRPA1 antagonist, had no effects on NaHS-induced scratching (Fig. 4D), wiping (Fig. 4E) or flinching (Fig. 4F). Together, these data suggest that neither TRPV1 nor TRPA1 is required for H_2_S donors-induced itch, while TRPV1 plays an important role in NaHS-induced spontaneous pain in mice.

### Activation of T-type calcium channels were indispensable H_2_S donors-induced itch in mice

**Figure 5:**
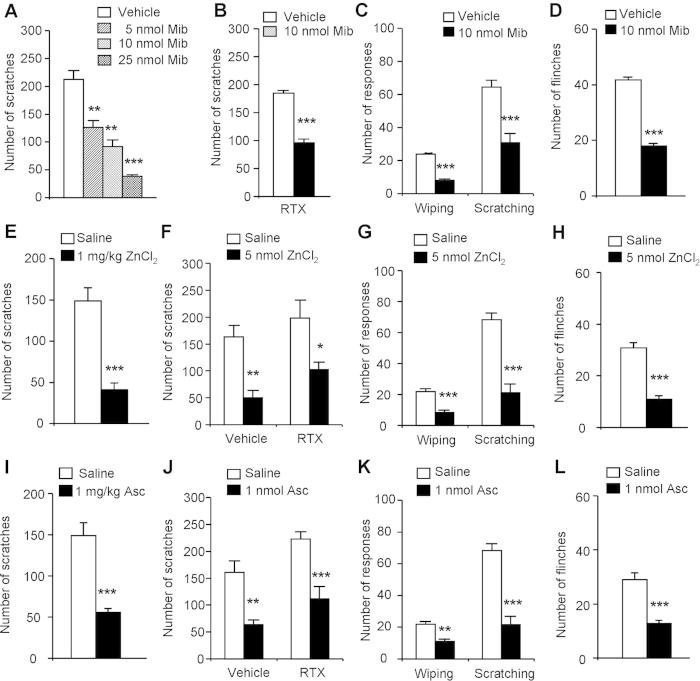
The inhibitory effects of T-type calcium channel blockers on NaHS-induced itch and pain behaviors in mice. (**A**) Systemic zinc chloride (ZnCl_2_; i.p. 1 mg/kg) significantly inhibited NaHS-induced scratching. (**B**) Local application of ZnCl_2_ (i.d. 5 nmol) significantly inhibited NaHS-induced scratching in both RTX- and vehicle-treated mice. (**C**) ZnCl_2_ (i.d. 5 nmol) significantly inhibited NaHS-induced both forelimb wiping and hindpaw scratching in cheek model. (**D**) ZnCl_2_ (i.pl. 5 nmol) significantly inhibited NaHS-induced flinching. (**E**) Systemic ascorbic acid (Asc; i.p. 1 mg/kg) significantly inhibited NaHS-induced scratching. (**F**) Asc (i.d. 1 nmol) significantly inhibited NaHS-induced scratching in both RTX- and vehicle-treated mice. (**G**) Asc (i.d. 1 nmol) significantly inhibited NaHS-induced both forelimb wiping and hindpaw scratching in cheek model. (**H**) Asc (i.pl. 1 nmol) significantly inhibited NaHS-induced flinching. (**I**) Local application of mibefradil (Mib) (i.d. 5–25 nmol) dose-dependently inhibited NaHS-induced scratching in mice. (**J**) Mib (i.d. 10 nmol) significantly inhibited NaHS-induced scratching in RTX-treated mice. (**K**) Mib (i.d. 10 nmol) significantly inhibited NaHS-induced both forelimb wiping and hindpaw scratching in cheek model. (**L**) Mib (i.d. 10 nmol) significantly inhibited NaHS-induced flinching. All data are expressed by means ± SEM. *n* = 6–8 mice per group. *P < 0.05; **P < 0.01, ***P < 0.001 vs. vehicle control, Student’s *t* test.

Previous reports showed that endogenous and exogenous H_2_S facilitates T-type calcium channel currents and contributes to pain sensation^38^,^39^,^40^,^41^. We thus investigated whether activation of T-type calcium channels contribute to NaHS-induced itch behaviors in mice. We found that local application of pan-T-type calcium channel blocker mibefradil (Mib) (i.d. 5–25 nmol) dose-dependently inhibited NaHS-induced scratching in naïve mice (Fig. 5A) and RTX-treated mice (184.4 ± 4.8 vs. 96.1 ± 6.7; *P* < 0.001; Fig. 5B). Mib (i.d. 10 nmol) significantly inhibited NaHS-induced forelimb wiping (24.0 ± 0.7 vs. 8.0 ± 0.9; *P* < 0.001; Fig. 5C) and hindpaw scratching (64.4 ± 4.3 vs. 30.7 ± 5.6; *P* < 0.001; Fig. 5C) in cheek model. Mib (i.d. 10 nmol) also significantly inhibited NaHS-induced flinching (41.8 ± 1.0 vs. 18.0 ± 0.9; *P* < 0.001; Fig. 5D). We further asked whether zinc chloride (ZnCl_2_) or ascorbic acid (Asc), two selectively inhibitors for Ca_v_3.2^42^, but not Ca_v_3.1 or Ca_v_3.3, isoforms of T-type calcium channels, affects NaHS-induced itch. Systemic (i.p. 1 mg/kg) and local application of ZnCl_2_ (i.d. 5 nmol) zinc chloride significantly inhibited NaHS-induced scratching in naïve (for i.p. injection: 148.8 ± 15.9 vs. 40.0 ± 0.6; *P* < 0.001; Fig. 5E; for i.d. injection: 162.8 ± 21.4 vs. 50.2 ± 13.5; *P* < 0.01; Fig. 5F) and RTX-treated mice (i.d. injection: 197.8 ± 33.9 vs. 103.0 ± 13.2; *P* < 0.05; Fig. 5F). In cheek model, ZnCl_2_ (i.d. 5 nmol) significantly inhibited NaHS-induced forelimb wiping (22.0 ± 1.7 vs. 8.4 ± 1.5; *P* < 0.001) and hindpaw scratching (68.2 ± 4.5 vs. 21.2 ± 5.5; *P* < 0.001; Fig. 5G). ZnCl_2_ (i.pl. 5 nmol) significantly inhibited NaHS-induced flinching (30.8 ± 2.1 vs. 11.0 ± 1.2; *P* < 0.001; Fig. 5H). Similarly, systemic (i.p. 1 mg/kg) and local application of ascorbic acid (Asc; i.d. 5 nmol) significantly inhibited NaHS-induced scratching in both naïve mice (for i.p. injection: 148.8 ± 14.2 vs. 55.8 ± 4.4; *P* < 0.001; Fig. 5I; for i.d. injection: 160.9 ± 21.5 vs. 63.8 ± 8.7; *P* < 0.01; Fig. 5J) and RTX-treated mice (i.d. injection: 223.2 ± 13.0 vs. 111.2 ± 23.0; *P* < 0.001; Fig. 5J). In cheek model, Asc (i.d. 1 nmol) significantly inhibited NaHS-induced forelimb wiping (22.5 ± 1.5 vs. 11.0 ± 1.7; *P* < 0.01) and hindpaw scratching (67.8 ± 3.7 vs. 21.7 ± 5.3; *P* < 0.001; Fig. 5K). Asc (i.pl. 1 nmol) significantly inhibited NaHS-induced flinching (29.0 ± 2.5 vs. 12.8 ± 1.1; *P* < 0.001; Fig. 5L). Together, these data indicated Ca_v_3.2 T-type calcium channels play key roles in H_2_S donors-induced itch, as well as pain sensation in mice.

### Silencing of Ca_v_3.2 T-type calcium channels in primary sensory neurons abolished NaHS-induced itch in mice

**Figure 6:**
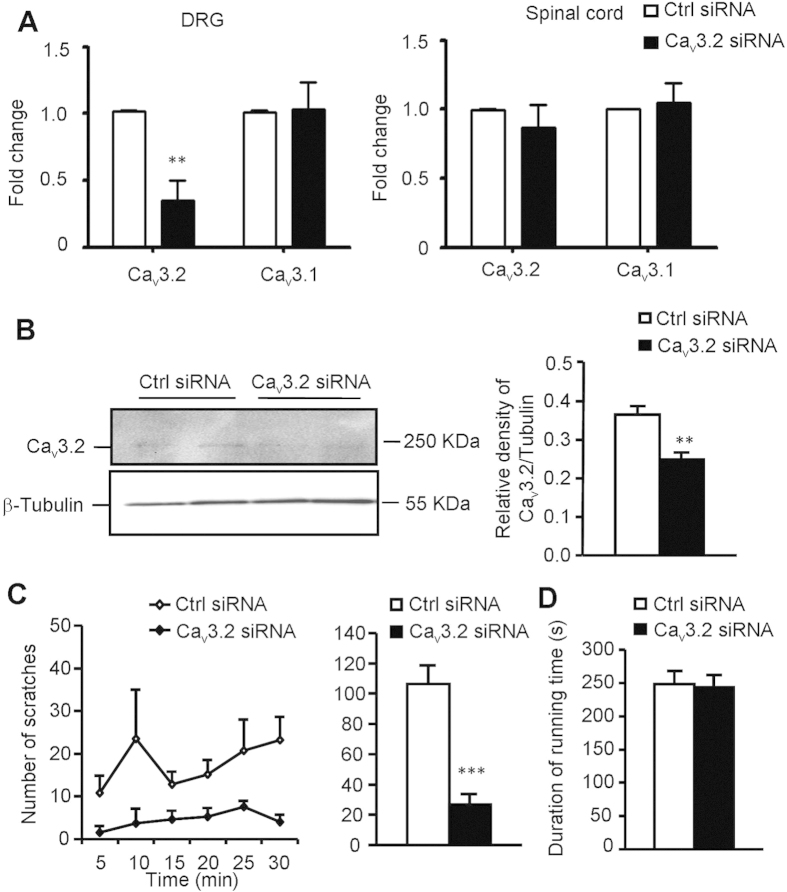
The inhibitory effects of silencing Ca_v_3.2 T-type calcium channel on NaHS-induced itch behaviors in mice. The mice received intrathecal (i.t.) injection of siRNA targeted Cav3.2 (2 μg/mice) or non-targeted control siRNA once a day for 2 days. (**A**) Q-PCR analysis showed that i.t. application of siRNA targeted Ca_v_3.2 selective knockdown the mRNA expression of Ca_v_3.2 channel, but not Ca_v_3.1 channel in DRG. In contrast, neither Ca_v_3.2 nor Ca_v_3.1 expression was affected in spinal cord. (**B**) Western blotting showed significant reduction of the Ca_v_3.2 expression at protein level. (**C**) Behavioral analysis showed that NaHS-induced scratching behavior was significantly inhibited by treatment of Ca_v_3.2-targeted siRNA. (**D**) Rotarod test showed normal motor function following Ca_v_3.2-targeted siRNA treatment. All data are expressed by means ± SEM. *n* = 6 mice per group. *P < 0.05; **P < 0.01, ***P < 0.001 vs. vehicle control, Student’s *t* test.

To avoid the nonspecific effects of pharmacological antagonists of T-type calcium channels, we used intrathecal injection of siRNA specific targeted to Ca_v_3.2 channel (Ca_v_3.2-siRNA) to knockdown its expression in primary sensory neurons in dorsal root ganalia (DRG). It was found that repeated intrathecal injection of Ca_v_3.2-siRNA, but not scrambled control siRNA, selective knockdown the expression of Ca_v_3.2, but not Ca_v_3.1 in DRG (Fig. 6A). Interestingly, the expression of Ca_v_3.2 in spinal cord was not affected by Ca_v_3.2-siRNA treatment (Fig. 6A), suggesting DRG is more accessible than spinal cord via intrathecal puncture. Western blotting also confirmed that Ca_v_3.2 protein expression in DRG was reduced by Ca_v_3.2-siRNA treatment (Fig. 6B). Behaviorally, NaHS-induced scratching was dramatically decreased in Ca_v_3.2-siRNA treated mice (106.3 ± 12.4 vs. 26.9 ± 6.9; *P* < 0.001; Fig. 6C). The motor function of Ca_v_3.2-siRNA treated mice was not affected (Fig. 6D). Thus, these data further emphasized the crucial roles of Ca_v_3.2 T-type calcium channels in H_2_S donors-induced itch in mice.

### Activation of T-type calcium channels was required for H_2_S donors-induced alloknesis (touch-evoked itch) in mice

**Figure 7:**
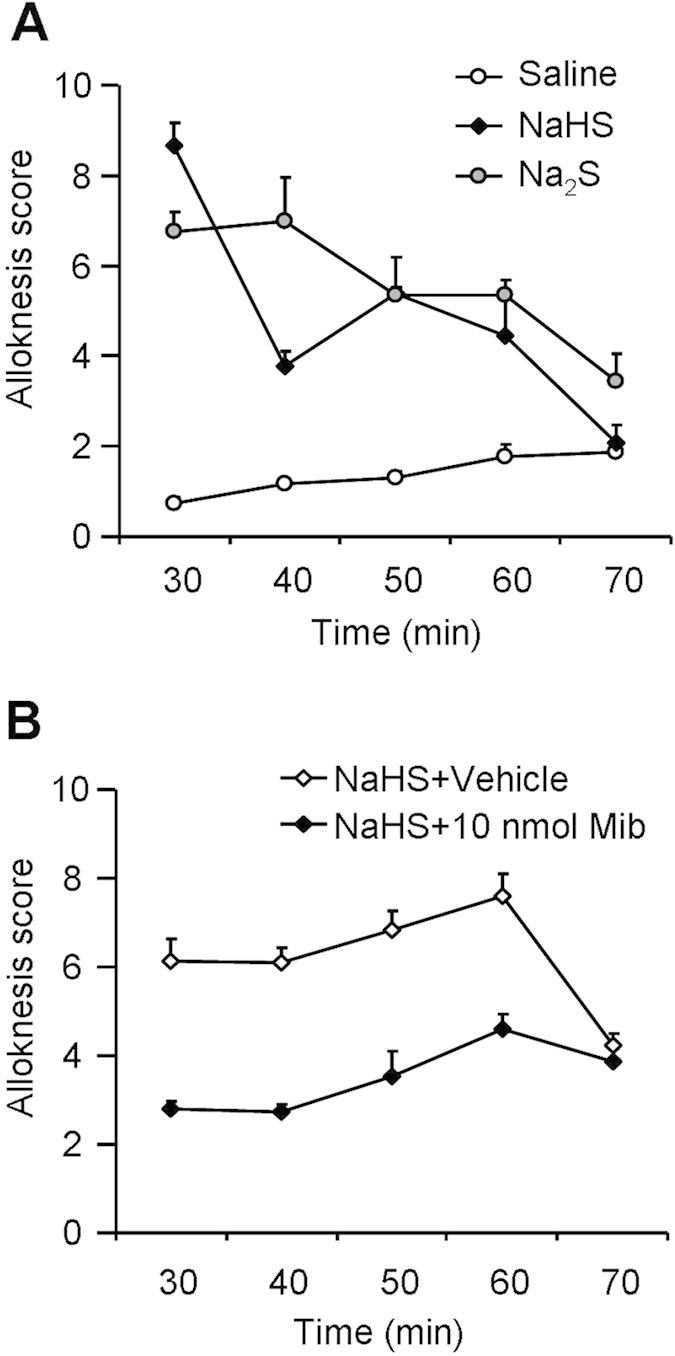
H_2_S donors induced alloknesis and T-type calcium channel blocker mibefradil suppressed this alloknesis in mice. (**A**) Time course of alloknesis induced by i.d. injection of H_2_S donors NaHS or Na_2_S in mice (*P* < 0.05). (**B**) Inhibitory effects of local injection of mibefradil on NaHS-induced alloknesis in mice (*P* < 0.05). All data are expressed by means ± SEM. Two-way repeated-measures ANOVA, *n* = 7 mice per group.

Alloknesis is a remarkable feature of chronic itch; however the mechanisms underlying this phenomenon are still unclear. Alloknesis was previously observed following i.d. histamine, 5-HT, protease-activated receptor (PAR)-4 agonist, and MrgprC11 agonist, but not chloroquine or a PAR-2 agonist, suggesting not all pruritogens evoked alloknesis. In this study, we asked whether H_2_S donors are able to induce alloknesis in mice. The results showed that i.d. NaHS or Na_2_S induced alloknesis in mice, which lasted for at least 30 min (Fig. 7A). Local co-administration of T-type calcium channels blocker Mib significantly suppressed the development of alloknesis elicited by NaHS (Fig. 7B).

### Involvement of endogenous H_2_S in compound 48/80-induced itch in mice

**Figure 8:**
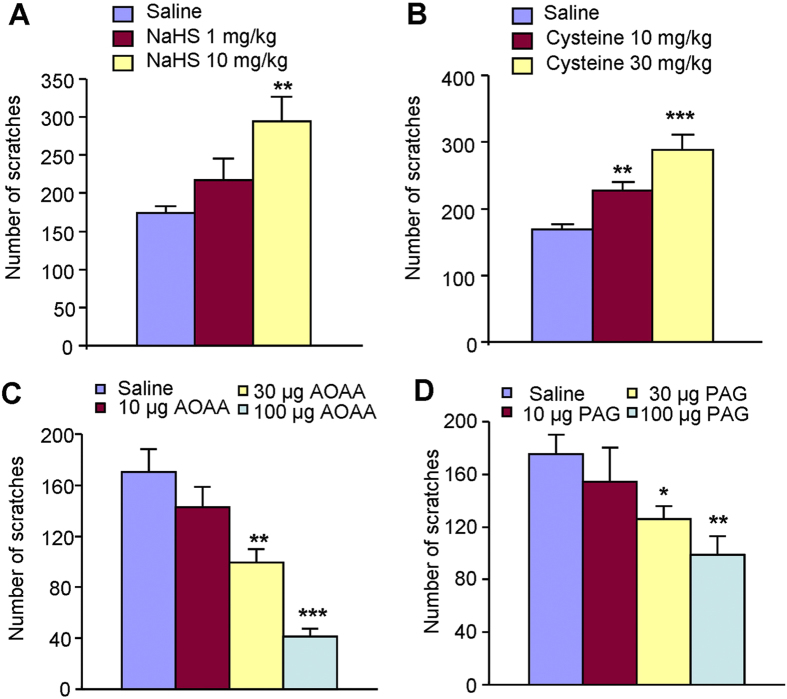
Endogenous H_2_S production contributes to compound 48/80-induced itch in mice. (**A**) Systemic administration of NaHS (1–10 mg/kg; i.p.) increased compound 48/80-induced scratching in mice. (**B**) Systemic administration of L-cysteine (10–30 mg/kg; i.p.) increased compound 48/80-induced scratching in a dose-dependent manner in mice. (**C**) Local administration of CBS inhibitor AOAA (10–100 μg; i.d.) dose-dependently attenuated compound 48/80-induced scratching in mice. (**D**) Local administration of CSE inhibitor PAG (10–100 μg; i.d.) dose-dependently attenuated compound 48/80-induced scratching in mice. All data are expressed by means ± SEM. *n* = 7–11 mice per group. *P < 0.05, **P < 0.01; ***P < 0.001 vs. control, one-way AVOVA following Bonferroni *post hoc* test.

We asked whether endogenous H_2_S is involved in itch responses induced by pruritogens in mice. Compound 48/80, a mast cell degranulator, is known to induce allergic itch via histamine release^43^. Systemic NaHS (10 mg/kg, i.p.) was able to enhanced compound 48/80-induced scratching behavior (Fig. 8A). Systemic administration of l-cystein (10–30 mg/kg, i.p.), a key precursor for endogenous H_2_S synthesis, significantly increased compound 48/80-induced scratching behavior in a dose-dependent manner in mice (Fig. 8B). Thus, increased endogenous production of H_2_S may be able to enhance compound 48/80-induced scratching behavior in mice. We further examined the possible effects of inhibitors of H_2_S-produing enzymes on itch sensation in mice. Strikingly, local application of the CBS inhibitor AOAA or CSE inhibitor PAG dose-dependently reduced compound 48/80-induced scratching behavior in mice (Fig. 8C,D), suggesting the inhibitors of endogenous H_2_S-produing enzymes are able to relief itch in mice. Together, endogenous H2S may be involved in allergic itch in mice.

**Figure 9:**
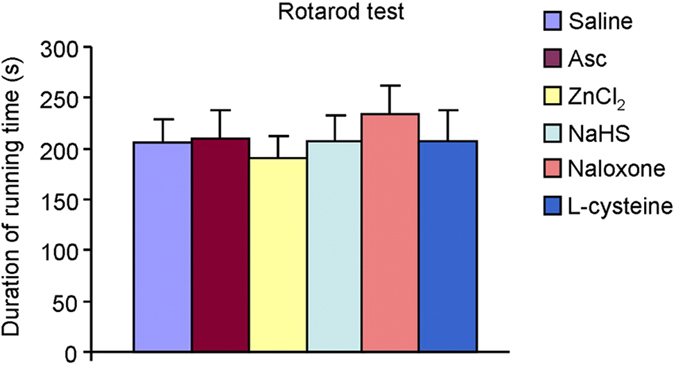
Rotarod testing showed the normal motor function following systemic administration of drugs, including naloxone (1 mg/kg), Asc (1 mg/kg), ZnCl2 (1 mg/kg), NaHS (1 mg/kg) and L-cysteine (10 mg/kg). None of these drugs affected the duration of running time in mice. *n *= 6 mice per group.

Finally, we tested the motor function following systemic administration of drugs used in this study. The results showed that systemic application of naloxone, Asc, ZnCl2, NaHS and L-cysteine did not affect the duration of running time using Rotarod test (Fig. 9), suggesting the effects of these drug on scratching behavior did not attribute to their influence on motor function.

## Discussion

Along with pain, touch and thermal sensation, itch is a cutaneous sensation and detected by primary sensory neurons in dorsal root ganglia (DRG) for the body and trigeminal ganglia (TG) for the face. Itch can be acute (e.g., mosquito bite) or chronic (e.g., atopic dermatitis). Acute itch may serve as a warning system, while chronic itch represents a common clinical problem^44^,^45^. Antihistamines are first line treatment for allergic itch; however, they are less efficient for many types of chronic itch^44^ (e.g. cholestasis, renal failure and apotic dermatitis), suggesting histamine-independent mechanisms are involved in. Great progress has been made in recent years to identify series of novel nonhistaminergic itch mediators^46^,^47^,^48^, such as PAR 2/4 agonists, MrgprA3/C11 agonists and TGR5 (also called Gpbar1) agonists. Interestingly, there were several reports that emphasized the crucial role of nitric oxide (NO) in itch elicited by chloroquine, serotonin and substance P^17^,^18^,^19^, indicating a key role of gasotransmitters in itch signaling. However, it is largely unknown about the roles of H_2_S, the third gasotransmitter, in itch regulation.

In the current study, we have identified the critical roles of H_2_S (the third gasotransmitter) in itch. We firstly observed that H_2_S donors (NaHS and Na_2_S) could elicit robust scratching behavior, which is μ-opioid receptor-dependent and histamine-independent in mice. Interestingly, slow-releasing H_2_S donor (GYY4137) was not able to elicit scratching in mice. As H_2_S donors are potential therapeutic for many diseases, slow-releasing H_2_S donors may be a good choice to avoid unwanted side effects, such as itch. We further revealed that activation of Ca_v_3.2 T-type calcium channel possible located in capsaicin-insensitive A-fibers, but not TRPV1 and TRPA1 in capsaicin-sensitive C-fibers, is required for H_2_S donors-elicited itch response. In contrast, activation of both Ca_v_3.2 T-type calcium channel and TRPV1 is essential for H_2_S-induced pain behavior^38^,^40^. We also demonstrated that endogenous H_2_S contributes to compound 48/80-induced itch sensation. Thus, our results identified H_2_S as a novel non-histaminergic itch mediator and provided several molecular targets for anti-itch treatment, such as T-type calcium channel inhibitors or H_2_S synthesis inhibitors.

Primary sensory neurons located in DRGs and TGs are responsible for itch signaling transmission from skin to spinal cord dorsal horn^1^,^44^,^49^. It is traditionally considered that TRPV1-expressing C-fibers, which include a subset of TRPA1-expressing neurons, are required for both histamine-dependent and independent itch^34^,^35^. In the present study, we surprisingly found that ablation of TRPV1-expressing C-fibers, which caused by systemic RTX treatment, did not affect H_2_S donors-induced itch, but abolished compound 48/80 or chloroquine-induced itch in mice (Fig. 3). Thus, TRPV1-expressing C-fibers are not essential for H_2_S donors-induced itch, although they were essential for H_2_S donors-induced pain behavior in mice. Local application of lidocaine (2%) completely abolished NaHS-induced itch in vehicle- and RTX-treated mice (Fig. 3), suggesting capsaicin-insensitive A-fibers may participate in NaHS-induced itch in mice. Previous reports clearly showed that small myelinated A-fibers mediated cowhage-induced histamine-independent itch in human^50^. In this study, we provided strong pharmacological evidence supporting that capsaicin-insensitive A-fibers mediate H_2_S donors-induced non-histaminergic itch, while capsaicin-sensitive C-fibers are essential for H_2_S donors-induced pain in mice. Consistently, TRPV1 and TRPA1 are dispensable for H_2_S donors-induced itch, although they might be important for H_2_S donors-induced pain in mice.

H_2_S signaling plays different physiological or pathological roles through acting on distinct receptors or channels^14^. We then asked which receptors H_2_S might act on for mediating itch sensation. Several ion channels or receptors had been identified as possible targets for H_2_S, such as TRPV1, TRPA1, T-type calcium channels and ATP-sensitive potassium channels etc^38^,^40^,^51^. Importantly, a recent study demonstrated that NaHS activated Ca_v_3.2 T-type calcium channels at higher concentration (3–10 mM); However, NaHS selective inhibited Ca_v_3.2 channels at μM concentration^52^. It indicated that the effects of NaHS on Ca_v_3.2 channels are dose-dependent. The doses used in the present study were mM concentration and NaHS at these doses were sufficient to activate Ca_v_3.2 channels. Consistently, inhibition or silencing of Ca_v_3.2 channels abolished NaHS-induced scratching in mice (Figs 5 and 6). To our knowledge, there is no report to directly measure the H_2_S level in/or around primary sensory neurons. Indeed, there were some reports showed the concentration of H_2_S in plasma ranged from 20–100 μM^53^. However, the local concentration of H_2_S may be high enough to activate Ca_v_3.2 channels under pathological condition, such as irritable bowel syndrome^54^ or acute H_2_S intoxication^55^. Acute exposure H_2_S also cause eye or skin irritation, including itching^55^. Base on the crucial roles of T-type calcium channels (especial Ca_v_3.2 channel) for itch, the relative lower concentration of H_2_S may suppress itch via its inhibition of Ca_v_3.2 channel under physiological condition. It needs further investigation on the H_2_S levels under physiological and pathological conditions and the roles of Ca_v_3.2 channel on chronic itch.

T-type calcium channels, including three difference types (termed Ca_v_3.1, Ca_v_3.2, and Ca_v_3.3), are low voltage activated and play important roles in electrical signaling in nerve, heart and muscle^56^. The isoform Ca_v_3.2 is predominant expressed in primary sensory neurons in DRG, which is activated at low voltage close to the resting membrane potentials and controls the bursting firing in sensory neurons and so potently modulates neuronal excitability^52^. Recent studies identified that Ca_v_3.2 is expressed by Aδ-low-threshold mechanoreceptors (LTMRs) and C-LTMRs and is required for light-touch perception and mechanical hypersensitivity (allodynia) under neuropathic pain^57^. Our results not only showed that Ca_v_3.2 mediated H_2_S donors-induced itch, but also demonstrated that Ca_v_3.2 contributes to NaHS-induced alloknesis (touch-evoked itch). Consistent with the cellular distribution of Ca_v_3.2 channel, Ca_v_3.2 more likely plays key role in mechanically-evoked itch. Although the phenomenon of mechanically-evoked itch had been observed in human^58^, the molecular basis for mechanically-evoked itch (a sub-modality of itch) is unclear. We provided important clues for roles of Ca_v_3.2 in mechanically-evoked itch, at least for NaHS-induced touch-evoked itch. The precise contribution of Ca_v_3.2 to mechanically-evoked itch warrants further investigation.

We finally asked about the roles of endogenous H_2_S in acute itch caused by compound 48/80. The results showed that increased endogenous production of H_2_S (i.p. injection of NaHS or L-cystein) is able to enhance compound 48/80-induced scratching behavior in mice (Fig. 8). Local application of the CBS inhibitor AOAA or CSE inhibitor PPG also dose-dependently reduced compound 48/80-induced scratching behavior in mice (Fig. 8). Although the expression changes of CBS or CSE under pathological itch conditions need further investigation, our data indicates that manipulating the level of endogenous production of H_2_S is able to modulate itch responses.

In summary, our findings showed that H_2_S donors-induced itch response is largely independent of histamine. H_2_S may activate Ca_v_3.2 T-type calcium channel possible in capsaicin-insensitive A-fibers, but not TRPV1 and TRPA1 in capsaicin-sensitive C-fibers, to elicit itch response in mice. Although further investigation is needed to reveal the roles of H_2_S in chronic itch, our findings strongly suggest that H_2_S is one of non-histaminergic itch mediators, although other gasotransmitter may also be involved in itch. Targeting H_2_S synthesis or Ca_v_3.2 T-type calcium channel may lead to the development of novel and effective anti-itch treatment.

## Additional Information

**How to cite this article**: Wang, X.-L. *et al.* Hydrogen sulfide-induced itch requires activation of Ca_v_3.2 T-type calcium channel in mice. *Sci. Rep.* **5**, 16768; doi: 10.1038/srep16768 (2015).

## References

[b1] Ikoma A., Steinhoff M., Stander S., Yosipovitch G. & Schmelz M. The neurobiology of itch. Nat. Rev. Neurosci. 7, 535–547 (2006). 16791143 10.1038/nrn1950

[b2] Han L. &Dong X. Itch mechanisms and circuits. Annu. Rev. Biophys. 43, 331–355 (2014). 24819620 10.1146/annurev-biophys-051013-022826 PMC4081479

[b3] Yosipovitch G. & Bernhard J. D. Clinical practice. Chronic pruritus. N. Engl. J. Med. 368, 1625–1634 (2013). 23614588 10.1056/NEJMcp1208814

[b4] Liu Q. *et al.* Sensory neuron-specific GPCR Mrgprs are itch receptors mediating chloroquine-induced pruritus. Cell 139, 1353–1365 (2009). 20004959 10.1016/j.cell.2009.11.034 PMC2989405

[b5] Davidson S., Zhang X., Khasabov S. G., Simone D. A. & Giesler G. J. Jr. Relief of itch by scratching: state-dependent inhibition of primate spinothalamic tract neurons. Nat. Neurosci. 12, 544–546 (2009). 19349977 10.1038/nn.2292 PMC3006451

[b6] Miller G. Biomedicine. Grasping for clues to the biology of itch. Science 318, 188–189 (2007). 17932266 10.1126/science.318.5848.188

[b7] Wang R. Two’s company, three’s a crowd: can H2S be the third endogenous gaseous transmitter? FASEB J. 16, 1792–1798 (2002). 12409322 10.1096/fj.02-0211hyp

[b8] Bos E. M., van G. H., Joles J. A., Whiteman M. & Leuvenink H. G. Hydrogen sulfide: physiological properties and therapeutic potential in ischaemia. Br. J. Pharmacol. 172, 1479–1493 (2015). 25091411 10.1111/bph.12869 PMC4369258

[b9] Du J. *et al.* Hydrogen sulfide suppresses oxidized low-density lipoprotein (ox-LDL)-stimulated monocyte chemoattractant protein 1 generation from macrophages via the nuclear factor kappaB (NF-kappaB) pathway. J. Biol. Chem. 289, 9741–9753 (2014). 24550391 10.1074/jbc.M113.517995 PMC3975021

[b10] Hu L. F., Lu M., Hon Wong P. T. & Bian J. S. Hydrogen sulfide: neurophysiology and neuropathology. Antioxid. Redox. Signal. 15, 405–419 (2011). 20812864 10.1089/ars.2010.3517

[b11] Kimura H. The physiological role of hydrogen sulfide and beyond. Nitric. Oxide. 41, 4–10 (2014). 24491257 10.1016/j.niox.2014.01.002

[b12] Liu Y. H., Yan C. D. & Bian J. S. Hydrogen sulfide: a novel signaling molecule in the vascular system. J. Cardiovasc. Pharmacol. 58, 560–569 (2011). 21283022 10.1097/FJC.0b013e31820eb7a1

[b13] Pouokam E. & Diener M. Mechanisms of actions of hydrogen sulphide on rat distal colonic epithelium. Br. J. Pharmacol. 162, 392–404 (2011). 20840536 10.1111/j.1476-5381.2010.01026.x PMC3031060

[b14] Smith H. S. Hydrogen sulfide’s involvement in modulating nociception. Pain Physician 12, 901–910 (2009). 19787017

[b15] Qi F. *et al.* Promoter demethylation of cystathionine-beta-synthetase gene contributes to inflammatory pain in rats. Pain 154, 34–45 (2013). 23273102 10.1016/j.pain.2012.07.031

[b16] Liu T. & Ji R. R. New insights into the mechanisms of itch: are pain and itch controlled by distinct mechanisms? Pflugers Arch. 465, 1671–1685 (2013). 23636773 10.1007/s00424-013-1284-2 PMC3796138

[b17] Andoh T. & Kuraishi Y. Nitric oxide enhances substance P-induced itch-associated responses in mice. Br. J. Pharmacol. 138, 202–208 (2003). 12522091 10.1038/sj.bjp.0705004 PMC1573631

[b18] Foroutan A., Haddadi N. S., Ostadhadi S., Sistany N. & Dehpour A. R. Chloroquine-induced scratching is mediated by NO/cGMP pathway in mice. Pharmacol. Biochem. Behav. 134, 79–84 (2015). 25957523 10.1016/j.pbb.2015.04.016

[b19] Ostadhadi S., Haj-Mirzaian A., Azimi E., Mansouri P. & Dehpour A. R. Involvement of nitric oxide in serotonin-induced scratching in mice. Clin. Exp. Dermatol. 40, 647–652 (2015). 25703534 10.1111/ced.12605

[b20] Shimada S. G. & LaMotte R. H. Behavioral differentiation between itch and pain in mouse. Pain 139, 681–687 (2008). 18789837 10.1016/j.pain.2008.08.002 PMC2723192

[b21] Akiyama T., Carstens M. I. & Carstens E. Facial injections of pruritogens and algogens excite partly overlapping populations of primary and second-order trigeminal neurons in mice. J. Neurophysiol. 104, 2442–2450 (2010). 20739601 10.1152/jn.00563.2010 PMC3350035

[b22] Xu Z. Z. *et al.* Resolvins RvE1 and RvD1 attenuate inflammatory pain via central and peripheral actions. Nat. Med. 16, 592–597 (2010). 20383154 10.1038/nm.2123 PMC2866054

[b23] Liu T., Xu Z. Z., Park C. K., Berta T. & Ji R. R. Toll-like receptor 7 mediates pruritus. Nat. Neurosci. 13, 1460–1462 (2010). 21037581 10.1038/nn.2683 PMC2991508

[b24] Akiyama T. *et al.* Mouse model of touch-evoked itch (alloknesis). J. Invest Dermatol. 132, 1886–1891 (2012). 22418875 10.1038/jid.2012.52 PMC3375351

[b25] Ramabadran K., Bansinath M., Turndorf H. & Puig M. M. Tail immersion test for the evaluation of a nociceptive reaction in mice. Methodological considerations. J. Pharmacol. Methods 21, 21–31 (1989). 2704245 10.1016/0160-5402(89)90019-3

[b26] Inan S., Dun N. J. & Cowan A. Inhibitory effect of lidocaine on pain and itch using formalin-induced nociception and 5′-guanidinonaltrindole-induced scratching models in mice: behavioral and neuroanatomical evidence. Eur. J. Pharmacol. 616, 141–146 (2009). 19549515 10.1016/j.ejphar.2009.06.026 PMC2735214

[b27] Berta T. *et al.* Transcriptional and functional profiles of voltage-gated Na( + ) channels in injured and non-injured DRG neurons in the SNI model of neuropathic pain. Mol. Cell Neurosci 37, 196–208 (2008). 17964804 10.1016/j.mcn.2007.09.007

[b28] Liu T. *et al.* TLR3 deficiency impairs spinal cord synaptic transmission, central sensitization, and pruritus in mice. J. Clin. Invest 122, 2195–2207 (2012). 22565312 10.1172/JCI45414 PMC3366391

[b29] Shimada S. G. & LaMotte R. H. Behavioral differentiation between itch and pain in mouse. Pain 139, 681–687 (2008). 18789837 10.1016/j.pain.2008.08.002 PMC2723192

[b30] Nojima H. *et al.* Opioid modulation of scratching and spinal c-fos expression evoked by intradermal serotonin. J. Neurosci. 23, 10784–10790 (2003). 14645470 10.1523/JNEUROSCI.23-34-10784.2003 PMC6740982

[b31] Liu X. Y. *et al.* Unidirectional Cross-Activation of GRPR by MOR1D Uncouples Itch and Analgesia Induced by Opioids. Cell 147, 447–458 (2011). 22000021 10.1016/j.cell.2011.08.043 PMC3197217

[b32] Shim W. S. & Oh U. Histamine-induced itch and its relationship with pain. Mol. Pain 4, 29 (2008). 18667087 10.1186/1744-8069-4-29 PMC2519061

[b33] Costa R. *et al.* Evidence for the role of neurogenic inflammation components in trypsin-elicited scratching behaviour in mice. Br. J. Pharmacol. 154, 1094–1103 (2008). 18454165 10.1038/bjp.2008.172 PMC2451046

[b34] Mishra S. K., Tisel S. M., Orestes P., Bhangoo S. K. & Hoon M. A. TRPV1-lineage neurons are required for thermal sensation. EMBO J. 30, 582–593 (2011). 21139565 10.1038/emboj.2010.325 PMC3034006

[b35] Imamachi N. *et al.* TRPV1-expressing primary afferents generate behavioral responses to pruritogens via multiple mechanisms. Proc. Natl. Acad. Sci. USA 106, 11330–11335 (2009). 19564617 10.1073/pnas.0905605106 PMC2708751

[b36] Wilson S. R. *et al.* TRPA1 is required for histamine-independent, Mas-related G protein-coupled receptor-mediated itch. Nat. Neurosci. 14, 595–602 (2011). 21460831 10.1038/nn.2789 PMC3181150

[b37] Lu W. *et al.* H2 S modulates duodenal motility in male rats via activating TRPV1 and K(ATP) channels. Br. J. Pharmacol. 171, 1534–1550 (2014). 24345161 10.1111/bph.12562 PMC3954491

[b38] Okubo K. *et al.* Hydrogen sulfide-induced mechanical hyperalgesia and allodynia require activation of both Ca_v_3.2 and TRPA1 channels in mice. Br. J. Pharmacol. 166, 1738–1743 (2012). 22300342 10.1111/j.1476-5381.2012.01886.x PMC3419915

[b39] Sekiguchi F. *et al.* Endogenous and exogenous hydrogen sulfide facilitates T-type calcium channel currents in Ca_v_3.2-expressing HEK293 cells. Biochem. Biophys. Res. Commun. 445, 225–229 (2014). 24508802 10.1016/j.bbrc.2014.01.185

[b40] Maeda Y. *et al.* Hyperalgesia induced by spinal and peripheral hydrogen sulfide: evidence for involvement of Ca_v_3.2 T-type calcium channels. Pain 142, 127–132 (2009). 19167819 10.1016/j.pain.2008.12.021

[b41] Xu G. Y. *et al.* The endogenous hydrogen sulfide producing enzyme cystathionine-beta synthase contributes to visceral hypersensitivity in a rat model of irritable bowel syndrome. Mol. Pain 5, 44 (2009). 19660142 10.1186/1744-8069-5-44 PMC2731739

[b42] Nelson M. T. *et al.* Reducing agents sensitize C-type nociceptors by relieving high-affinity zinc inhibition of T-type calcium channels. J. Neurosci. 27, 8250–8260 (2007). 17670971 10.1523/JNEUROSCI.1800-07.2007 PMC6673068

[b43] Schlosburg J. E., Boger D. L., Cravatt B. F. & Lichtman A. H. Endocannabinoid modulation of scratching response in an acute allergenic model: a new prospective neural therapeutic target for pruritus. J. Pharmacol. Exp. Ther. 329, 314–323 (2009). 19168707 10.1124/jpet.108.150136 PMC2670585

[b44] Paus R., Schmelz M., Biro T. & Steinhoff M. Frontiers in pruritus research: scratching the brain for more effective itch therapy. J. Clin. Invest 116, 1174–1186 (2006). 16670758 10.1172/JCI28553 PMC1451220

[b45] Patel K. N. & Dong X. Itch: Cells, Molecules, and Circuits. ACS Chem. Neurosci. 2, 17–25 (2011). 21720568 10.1021/cn100085g PMC3123905

[b46] Sikand P., Dong X. & LaMotte R. H. BAM8-22 peptide produces itch and nociceptive sensations in humans independent of histamine release. J. Neurosci. 31, 7563–7567 (2011). 21593341 10.1523/JNEUROSCI.1192-11.2011 PMC3111068

[b47] Reddy V. B., Iuga A. O., Shimada S. G., LaMotte R. H. & Lerner E. A. Cowhage-evoked itch is mediated by a novel cysteine protease: a ligand of protease-activated receptors. J. Neurosci. 28, 4331–4335 (2008). 18434511 10.1523/JNEUROSCI.0716-08.2008 PMC2659338

[b48] Liu Q. *et al.* The distinct roles of two GPCRs, MrgprC11 and PAR2, in itch and hyperalgesia. *Sci*. Signal. 4, ra45 (2011). 10.1126/scisignal.2001925 PMC3144551 21775281

[b49] Liu Y. & Ma Q. Generation of somatic sensory neuron diversity and implications on sensory coding. Curr. Opin. Neurobiol. 21, 52–60 (2011). 20888752 10.1016/j.conb.2010.09.003 PMC3029488

[b50] Ringkamp M. *et al.* A role for nociceptive, myelinated nerve fibers in itch sensation. J. Neurosci. 31, 14841–14849 (2011). 22016517 10.1523/JNEUROSCI.3005-11.2011 PMC3218799

[b51] Distrutti E. *et al.* Hydrogen sulphide induces micro opioid receptor-dependent analgesia in a rodent model of visceral pain. Mol. Pain 6, 36 (2010). 20540729 10.1186/1744-8069-6-36 PMC2908066

[b52] Elies J. *et al.* Hydrogen sulfide inhibits Ca_v_3.2 T-type Ca2 + channels. FASEB J. 28, 5376–5387 (2014). 25183670 10.1096/fj.14-257113

[b53] Peers C., Bauer C. C., Boyle J. P., Scragg J. L. & Dallas M. L. Modulation of ion channels by hydrogen sulfide. Antioxid. Redox. Signal. 17, 95–105 (2012). 22074224 10.1089/ars.2011.4359

[b54] Jimenez M. Hydrogen sulfide as a signaling molecule in the enteric nervous system. Neurogastroenterol. Motil. 22, 1149–1153 (2010). 21069935 10.1111/j.1365-2982.2010.01600.x

[b55] Reiffenstein R. J., Hulbert W. C. & Roth S. H. Toxicology of hydrogen sulfide. Annu. Rev. Pharmacol. Toxicol. 32, 109–134 (1992). 1605565 10.1146/annurev.pa.32.040192.000545

[b56] Todorovic S. M. & Jevtovic-Todorovic V. Targeting of Ca_v_3.2 T-type calcium channels in peripheral sensory neurons for the treatment of painful diabetic neuropathy. Pflugers Arch. 466, 701–706 (2014). 24482063 10.1007/s00424-014-1452-z

[b57] Francois A. *et al.* The Low-Threshold Calcium Channel Ca_v_3.2 Determines Low-Threshold Mechanoreceptor Function. Cell Rep. 10, 370–382 (2015). 10.1016/j.celrep.2014.12.042 25600872

[b58] Fukuoka M., Miyachi Y. & Ikoma A. Mechanically evoked itch in humans. Pain 154, 897–904 (2013). 23582153 10.1016/j.pain.2013.02.021

