# Corrigendum: Hydrogen sulfide-induced itch requires activation of Ca_v_3.2 T-type calcium channel in mice

**DOI:** 10.1038/srep46906

**Published:** 2017-10-20

**Authors:** Xue-Long Wang, Bin Tian, Ya Huang, Xiao-Yan Peng, Li-Hua Chen, Jun-Cheng Li, Tong Liu

Scientific Reports 5: Article number: 16768; 10.1038/srep16768 published online: 11 25 2015; updated: 10 20 2017.

This Article contains errors in the legend of Figure 5.

“(**A**) Systemic zinc chloride (ZnCl_2_; i.p. 1 mg/kg) significantly inhibited NaHS-induced scratching. (**B**) Local application of ZnCl_2_ (i.d. 5 nmol) significantly inhibited NaHS-induced scratching in both RTX- and vehicle-treated mice. (**C**) ZnCl_2_ (i.d. 5 nmol) significantly inhibited NaHS-induced both forelimb wiping and hindpaw scratching in cheek model. (**D**) ZnCl_2_(i.pl. 5 nmol) significantly inhibited NaHS-induced flinching. (**E**) Systemic ascorbic acid (Asc; i.p. 1 mg/kg) significantly inhibited NaHS-induced scratching. (**F**) Asc (i.d. 1 nmol) significantly inhibited NaHS-induced scratching in both RTX- and vehicle-treated mice. (**G**) Asc (i.d. 1 nmol) significantly inhibited NaHS-induced both forelimb wiping and hindpaw scratching in cheek model. (**H**) Asc (i.pl. 1 nmol) significantly inhibited NaHS-induced flinching. (**I**) Local application of mibefradil (Mib) (i.d. 5–25 nmol) dose-dependently inhibited NaHS-induced scratching in mice. (**J**) Mib (i.d. 10 nmol) significantly inhibited NaHS-induced scratching in RTX-treated mice. (**K**) Mib (i.d. 10 nmol) significantly inhibited NaHS-induced both forelimb wiping and hindpaw scratching in cheek model. (**L**) Mib (i.d. 10 nmol) significantly inhibited NaHS-induced flinching. All data are expressed by means ± SEM. *n* = 6–8 mice per group. *P < 0.05; **P < 0.01, ***P < 0.001 vs. vehicle control, Student’s *t* test”.

should read:

“(**A**) Local application of mibefradil (Mib) (i.d. 5–25 nmol) dose-dependently inhibited NaHS-induced scratching in mice. (**B**) Mib (i.d. 10 nmol) significantly inhibited NaHS-induced scratching in RTX-treated mice. (**C**) Mib (i.d. 10 nmol) significantly inhibited NaHS-induced both forelimb wiping and hindpaw scratching in cheek model. (**D**) Mib (i.d. 10 nmol) significantly inhibited NaHS-induced flinching. (**E**) Systemic zinc chloride (ZnCl2; i.p. 1 mg/kg) significantly inhibited NaHS-induced scratching. (**F**) Local application of ZnCl2 (i.d. 5 nmol) significantly inhibited NaHS-induced scratching in both RTX- and vehicle-treated mice. (**G**) ZnCl2 (i.d. 5 nmol) significantly inhibited NaHS-induced both forelimb wiping and hindpaw scratching in cheek model. (**H**) ZnCl2 (i.pl. 5 nmol) significantly inhibited NaHS-induced flinching. (**I**) Systemic ascorbic acid (Asc; i.p. 1 mg/kg) significantly inhibited NaHS-induced scratching. (**J**) Asc (i.d. 1 nmol) significantly inhibited NaHS-induced scratching in both RTX- and vehicle-treated mice. (**K**) Asc (i.d. 1 nmol) significantly inhibited NaHS-induced both forelimb wiping and hindpaw scratching in cheek model. (**L**) Asc (i.pl. 1 nmol) significantly inhibited NaHS-induced flinching. All data are expressed by means ± SEM. *n* = 6–8 mice per group. *P < 0.05; **P < 0.01, ***P < 0.001 vs. vehicle control, Student’s *t* test”.

